# Epigenetic safety of in vitro maturation in PCOS: genome-wide DNA methylation profiling of cord blood from a randomized controlled trial

**DOI:** 10.1186/s13048-026-02179-7

**Published:** 2026-06-27

**Authors:** Wei Guo, Yi Yang, Meng Qin, Siming Kong, Linlin Wang, Xiaoying Zheng, Rui Yang, Shuo Yang, Xiumei Zhen, Xiaohui Zhu, Rong Li, Liying Yan, Jie Qiao, Zhiqiang Yan

**Affiliations:** 1 Department of Obstetrics and Gynecology, State Key Laboratory of Female Fertility Promotion, Center for Reproductive Medicine, Peking University Third Hospital, No. 49 HuaYuan North Road, HaiDian District, Beijing, 100191 The People’s Republic of China; 2 National Clinical Research Center for Obstetrics and Gynecology, Peking University Third Hospital, Beijing, 100191 China; 3 Key Laboratory of Assisted Reproduction, Peking University, Ministry of Education, Beijing, 100191 China; 4 Beijing Key Laboratory of Reproductive Endocrinology and Assisted Reproductive Technology, Beijing, 100191 China; 5 Department of Obstetrics and Gynecology, Centre for Reproductive Medicine, Peking University Third Hospital, Beijing, 100191 China; 6 Research Units of Comprehensive Diagnosis and Treatment of Oocyte Maturation Arrest, Chinese Academy of Medical Sciences, Beijing, 100191 China

**Keywords:** Polycystic Ovary Syndrome (PCOS), In Vitro Maturation (IVM), In Vitro Fertilization (IVF), Assisted Reproductive Technology (ART), Umbilical Cord Blood (UCB), DNA methylation, Epigenetics safety

## Abstract

**Background:**

*In vitro* maturation (IVM) provides a safer alternative to conventional *in vitro* fertilization (IVF) for women with polycystic ovary syndrome (PCOS) by mitigating the risk of ovarian hyperstimulation. However, concerns persist regarding whether IVM perturbs epigenetic reprogramming in the offspring. Current evidence is constrained by candidate-gene approaches or a lack of parental controls. This study aimed to evaluate the genome-wide DNA methylation safety of IVM compared with conventional IVF using a rigorous trio-based design.

**Methods:**

This secondary epigenetic analysis was nested within a randomized controlled trial (RCT) (ClinicalTrials.gov: NCT03463772). We included 10 nuclear families (trios), comprising five IVM-conceived and five IVF-conceived singleton offspring alongside their biological parents. Both groups utilized a uniform freeze-only single-blastocyst transfer strategy to minimize hormonal confounding.

Genomic DNA from umbilical cord blood (UCB) and parental peripheral blood was analyzed using reduced representation bisulfite sequencing (RRBS). Genome-wide methylation patterns and differentially methylated regions (DMRs) were subsequently compared between the groups.

**Results:**

Clinical characteristics were comparable between the IVM and IVF groups.

Genome-wide analyses demonstrated high concordance in UCB methylation patterns, revealing no significant differences in global CpG methylation levels or distributions across key genomic features (promoters, CpG islands, and gene bodies). Only three rare DMRs were identified in UCB (representing ~ 0.0001% of the genome), none of which mapped to imprinted or developmentally critical loci. Furthermore, methylation variability remained consistent between the groups.

**Conclusions:**

Our findings provide robust mechanistic evidence supporting the epigenetic safety of IVM. The remarkable stability of the neonatal methylome confirms that specific IVM conditions do not compromise early developmental programming, thereby endorsing IVM as a safe and viable alternative for women with PCOS.

**Trial registration:**

ClinicalTrials.gov registry, NCT03463772. Registered on March 13, 2018.

**Supplementary Information:**

The online version contains supplementary material available at https://doi.org/10.1186/s13048-026-02179-7.

## Introduction

Polycystic ovary syndrome (PCOS) is a heterogeneous endocrine–metabolic disorder affecting approximately 5–15% of women of reproductive age and remains a primary cause of anovulatory infertility [1–3]. In most clinical settings, PCOS is diagnosed according to the Rotterdam criteria, requiring at least two of the following: hyperandrogenism, oligo-/anovulation, and polycystic ovarian morphology after exclusion of related disorders [4]. In addition to reproductive dysfunction, PCOS is frequently accompanied by insulin resistance, obesity, and dyslipidemia. It is also associated with increased risks of pregnancy complications, including gestational diabetes and hypertensive disorders, which may profoundly influence the intrauterine environment experienced by the developing fetus [3, 4].

Conventional *in vitro* fertilisation (IVF) with controlled ovarian hyperstimulation (COH) is widely utilized to treat infertility in women with PCOS; however, this population often exhibits heightened gonadotropin sensitivity and subsequently faces an elevated risk of ovarian hyperstimulation syndrome (OHSS) [5]. *In vitro* maturation (IVM) provides a vital alternative strategy wherein immature oocytes are retrieved from unstimulated or minimally stimulated ovaries and matured ex vivo before fertilization. This approach reduces exposure to supraphysiological stimulation and largely circumvents OHSS [6, 7]. By alleviating treatment-related complications, reducing the associated physical burden, and potentially easing patient distress during high-responder cycles, IVM serves as an attractive option for selected women with PCOS. Nevertheless, because IVM fundamentally alters the maturation environment of the oocyte, concerns regarding the long-term safety of the offspring persist [7–9].

The specific timing of IVM—where oocytes are retrieved from small follicles and matured *in vitro*—coincides with the critical window of epigenetic reprogramming and imprint establishment. This raises theoretical concerns that the *in vitro* culture environment, which differs distinctly from in vivo follicular fluid, could perturb DNA methylation acquisition. This biological uncertainty presents a significant clinical gap: despite the clear benefit of eliminating OHSS, the lack of high-resolution epigenetic safety data acts as a barrier to the widespread adoption of IVM, leaving clinicians without a definitive safety profile to counsel high-risk PCOS patients.

Assisted reproductive technologies (ART) involve multiple interventions—ovarian stimulation, gamete handling, fertilisation procedures, and embryo culture—that occurring during a period of extensive epigenetic reprogramming in the oocyte and early embryo [10–12]. DNA methylation is a central regulator of gene expression and is essential for genomic imprinting and early development. Abnormal methylation at imprinted loci contributes to imprinting disorders and adverse developmental outcomes [13–15]. Furthermore, the periconceptional environment can influence methyl-group availability and one-carbon metabolism, providing a biologically plausible link between ART exposures and subtle epigenetic variation [16, 17]. Recent methylome profiling of human *in vitro* matured oocytes has delineated DNA methylation landscapes in IVM oocytes, providing a mechanistic context for assessing potential downstream effects on embryo development and offspring tissues [18].

Population-based studies comparing ART-conceived and naturally conceived children have generally reported modest, locus-specific methylation differences in neonatal tissues, typically with small effect sizes and limited consistency across cohorts, suggesting that widespread disruption of the methylome is uncommon [14, 19, 20].

Importantly, PCOS itself has been linked to epigenetic variation in maternal and offspring tissues, complicating the direct attribution of methylation differences solely to ART procedures [21, 22]. Comparative genome-wide evidence specifically evaluating IVM versus conventional IVF remains limited but is largely reassuring.

This includes an EPIC array study comparing CAPA-IVM with IVF across neonatal tissues [3] and follow-up data indicating comparable early childhood development up to 24 months after CAPA-IVM versus IVF in a randomised trial [23].

To address the remaining gap in genome-wide methylation evidence specifically comparing IVM versus conventional IVF within a PCOS population, we conducted a trio-based epigenetic sub-study nested within our previously published non-inferiority RCT [7]. Leveraging the specific cohort from that study (ClinicalTrials.gov: NCT03463772), we profiled umbilical cord blood (UCB) DNA methylation in offspring conceived through IVM versus conventional IVF using reduced representation bisulfite sequencing (RRBS). By utilizing parent–offspring trios and applying the same uniform ICSI and freeze-only single-blastocyst transfer strategy in both arms (as described in the primary RCT), this design significantly reduces the variability introduced by downstream laboratory and transfer practices, thereby enhancing the control for familial genetic background. We hypothesized that, within this PCOS cohort, IVM would not be associated with widespread or clinically meaningful alterations in the neonatal cord blood DNA methylome compared with conventional IVF. Validating this hypothesis would yield the necessary molecular reassurance to position IVM as a safe, first-line alternative for women with PCOS, enabling the prevention of OHSS without compromising the long-term epigenetic health of the offspring.

## Materials and methods

### Study design

This study is an ancillary epigenetic analysis nested within the parent open-label, non-inferiority RCT (Registry: ClinicalTrials.gov; Trial registration number: NCT03463772; Date of registration: March 13, 2018). For the present RRBS analysis, inclusion was strictly determined by the availability of high-quality biospecimens to ensure technical robustness. Specifically, inclusion required: (1) the complete availability of trio biospecimens (umbilical cord blood from the newborn and peripheral blood from both parents); (2) sufficient DNA quantity and quality for library preparation; and (3) the successful generation of sequencing libraries meeting predefined quality-control criteria.

Given that trio-complete biospecimen collection at delivery (particularly paternal sampling) is not universally obtainable in routine clinical workflows, and high-depth genome-wide methylation profiling is highly resource-intensive, the final dataset comprised 10 nuclear families (five IVM and five IVF). Crucially, this selection was conducted blindly regarding the pregnancy course, neonatal health outcomes, or downstream methylation results, thereby minimizing potential selection bias. While the sample size is limited compared to large epidemiological cohorts, employing a high-resolution trio-based design provides granular, mechanistic insights into inheritance patterns that cross-sectional studies cannot achieve, serving as a high-quality foundational dataset for future large-scale validations.

Eligible women were aged 20–38 years, had a diagnosis of PCOS according to the 2003 Rotterdam criteria (hyperandrogenism and/or ovulatory dysfunction plus polycystic ovarian morphology on ultrasound), and were undergoing their first oocyte retrieval cycle. The trial was approved by the Ethics Committee of Peking University Third Hospital (2017sz-066). The study design and protocol have been described in detail previously [7]. The reporting of this study adheres to the Consolidated Standards of Reporting Trials (CONSORT) guidelines. A completed CONSORT checklist is provided as an additional supplementary file.

All procedures were conducted in accordance with the Declaration of Helsinki and its subsequent amendments, and adhered to good clinical practice guidelines. Written informed consent was obtained from all participating women and their partners prior to enrollment in the RCT. For the epigenetic sub-study, additional informed consent for the collection and molecular analysis of UCB was obtained from the parents on behalf of their newborns, and peripheral blood samples were collected from both parents. Multiple pregnancies, the use of donor gametes, preimplantation genetic testing, and all newborns included in this analysis were confirmed to be free of major congenital anomalies.

### Ovarian stimulation, IVM/IVF procedures and embryo transfer

#### IVM group

The IVM protocol has been detailed previously. Women in the IVM arm did not receive gonadotrophins or human chorionic gonadotrophin (hCG) priming. The retrieval of immature oocytes was scheduled once the endometrial thickness reached at least 6 mm and the largest follicle measured < 10 mm in diameter. After transvaginal aspiration, all cumulus–oocyte complexes (COCs) were transferred into an IVM media kit (Sage, Trumbull, CT, USA) supplemented with 5 mg/mL serum protein substitute and MENOPUR (Menopur; Ferring, Kiel, Germany) and cultured in a 5% CO₂incubator at 37℃. The final concentrations in the IVM culture system were 0.075 IU/mL FSH and 0.010 IU/mL hCG. After 28–32 h of culture, cumulus cells were removed, and oocyte maturation status was assessed. All metaphase II (MII) oocytes were fertilised by intracytoplasmic sperm injection (ICSI). 

#### Conventional IVF group

Women in the conventional IVF group underwent a flexible gonadotropin-releasing hormone (GnRH) antagonist protocol. Recombinant FSH (rFSH; Gonal-F, Serono, Aubonne, Germany), with a starting dose of 112.5–225 IU, was administered from day 2 or 3 of the menstrual cycle. A GnRH antagonist was initiated once a dominant follicle reached 12–14 mm in diameter. When at least two leading follicles reached 17 mm, 250 µg recombinant hCG (rhCG; Ovidrel, Serono, Aubonne, Germany) was administered to trigger final oocyte maturation. Oocyte retrieval was performed 36 ± 2 h after triggering under intravenous sedation. Conventional IVF or ICSI was used for fertilisation according to semen parameters. 

#### Embryo freezing and transfer

A freeze-only blastocyst transfer strategy was implemented in both groups. Embryos were cultured to the blastocyst stage following standard laboratory procedures, and all viable blastocysts were vitrified using standard cryopreservation methods.

Endometrial preparation for frozen–thawed embryo transfer was identical in both the IVM and IVF arms. Oral estradiol valerate (Valiera, Laboratorios Recalcine SA, Puente Alto, Chile) 3 mg twice daily was started on day 2 of the menstrual cycle.

After a minimum of 10 days of estradiol treatment and once endometrial thickness reached ≥ 8 mm, vaginal progesterone gel (Crinone, Merck Serono, Watford, UK) 90 mg once daily and oral dydrogesterone (Duphaston, Abbott) 20 mg twice daily were added. Ultrasound monitoring was performed using a Voluson E8 digital platform. A single blastocyst was transferred on day 7 after initiation of progesterone.

Luteal phase support was continued until 12 weeks of gestation. The clinical characteristics of women undergoing IVM or conventional IVF, as well as pregnancy monitoring and delivery outcomes, were prospectively recorded and archived in the hospital database. 

### DNA extraction and Reduced Representation bBisulfite Sequencing (RRBS)

Genomic DNA (gDNA) was extracted from parental peripheral blood and UCB using the QIAmp DNA Blood Mini Kit (Qiagen Cat# 51,106), according to the manufacturer’s protocol [24]. Extracted DNA was stored at − 80℃ until downstream analysis.

RRBS libraries were generated following a previously published protocol with minor modifications [24, 25]. In brief, approximately 0.3% unmethylated lambda DNA (dam⁻; dcm⁻; Thermo Scientific, Cat. SD0021) was spiked into 500 ng of high-quality gDNA for each sample. The mixture was subsequently digested with the restriction enzyme MspI (Thermo Scientific, Cat. ER0541). Digested fragments were purified using Agencourt AMPure XP beads (Beckman Coulter, Cat. A63881), after which the DNA ends were repaired and A-tailed with Klenow Fragment (exo⁻) (Thermo Scientific, Cat. EP0422). Next, NEBNext adapters (New England Biolabs, Cat. E7335) were ligated to the A-tailed fragments overnight using T4 DNA ligase at high concentration (NEB, Cat. M0202M). The adapter-ligated products were subsequently treated with Uracil-Specific Excision Reagent (USER) enzyme (NEB, Cat. M5505L) to remove uracil-containing sequences. Bisulfite conversion was performed using the MethylCode Bisulfite Conversion Kit (Thermo Scientific, Cat. MECOV-50), according to the manufacturer’s instructions. Following bisulfite treatment, converted DNA fragments were size-selected on a 2% TAE agarose gel, and gel slices corresponding to DNA fragments of approximately 160–700 bp were excised. DNA was recovered from the gel using a commercial gel extraction kit (VISTECH, Cat. PC0313). The purified, size-selected fragments were then amplified by PCR using KAPA HiFi U+ Master Mix (Kapa Biosystems, Cat. KK2801) for up to 12 cycles, during which sample-specific barcodes were introduced. The amplified libraries were purified and quantified with a Qubit 3.0 Fluorometer using the Qubit dsDNA High Sensitivity Assay (Thermo Scientific, Cat. Q33216/Q32854). Fragment size distributions were evaluated on a Fragment Analyzer™ Automated CE System (Advanced Analytical Technologies; Analysis Kit, Cat. DNF-474-0500), and molar concentrations were determined with the NEBNext Library Quant Kit for Illumina (NEB, Cat. E7630L). Libraries that passed quality control were sequenced in paired-end 150-bp mode (PE150) on an Illumina X Ten platform.

### Data processing and alignment

Raw FASTQ files derived from DNA methylation sequencing were subjected to quality control using Trim Galore!. This process involved removal of random primer sequences, Illumina adapter sequences, low-quality bases and reads shorter than 50 bp. Following quality control, processed reads were aligned to the human reference genome (hg38) in single-end mode using Bismark with the parameter “-bowtie2”.

Subsequently, single-end alignment files were merged with Samtools and only uniquely aligned reads were retained for downstream analysis. Methylation site identification was performed using the bismark_methylation_extractor function in Bismark. The numbers of reads supporting cytosine (methylated) and thymine (unmethylated) at each CpG site were quantified based on sequence coverage.

### RRBS data analysis

Bisulphite conversion efficiency was estimated by aligning processed sequencing reads to the lambda DNA genome. It was calculated as the ratio of unmethylated cytosines (Cs) detected within the lambda genome coverage region to the total number of Cs present in that region. DNA methylation calling was based on bisulphite-induced C-to-T conversion: cytosines that remained as C after bisulphite treatment were interpreted as methylated, whereas cytosines converted to thymine (T) were considered unmethylated.

### Fetal methylation evaluation

DNA methylation levels and variability were evaluated for annotated genomic regions in UCB and parental peripheral blood (maternal peripheral blood, MPB; paternal peripheral blood, PPB) samples from both IVF and IVM groups. Given the uneven distribution of CpG sites across the human genome, we divided the genome into non-overlapping 500 bp tiles as units for genome-wide methylation profiling. The methylation level of each 500 bp tile was calculated by averaging the methylation values of all CpG sites within the tile. Samples were stratified by sample type (UCB/MPB/PPB) within the IVF and IVM groups for comparative analysis. To ensure data reliability, only tiles covered across all 10 samples were retained. Pairwise Pearson correlation coefficients between samples were then calculated using the cor function in R with the parameter pairwise.complete.obs.

### DMR analysis

In the analysis of DMRs, CpG sites were required to be covered in the majority of samples within the same sample type because of the two-group design of the study.

For each pairwise comparison within a given sample type between the IVM and IVF groups, only sites meeting the following criteria were retained: at least three samples per group with coverage of at least five reads at CpG sites on autosomal chromosomes. DMR identification was then performed using the methylKit R package with thresholds set to an absolute methylation difference ≥ 20% and q-value ≤ 0.05.

Only DMRs that were repeatedly identified in at least three pairwise comparisons between the IVM group (UCB/MPB/PPB) and the corresponding IVF group were ultimately considered valid. All identified DMRs were annotated using HOMER with default parameters.

### Statistical analysis

Continuous variables are presented as means ± standard deviations (SD) or medians (interquartile ranges), as appropriate. Continuous variables were compared using unpaired t-tests or one-way ANOVA. Levene’s test was used to evaluate the homogeneity of variances between the IVM and conventional IVF groups. 

Categorical variables were compared using the χ² test or Fisher’s exact test, as appropriate. For genome-wide methylation data, P-values were adjusted for multiple testing where applicable. All statistical analyses were performed using R (version 3.3.3; R Foundation for Statistical Computing, Vienna, Austria). A two-sided p-value < 0.05 was considered statistically significant.

## Results

### High concordance of genome-wide methylation profiles between offspring from IVM and IVF

To evaluate the impact of different ARTs on offspring DNA methylation, we performed RRBS on UCB samples from 10 neonates (five conceived by IVM and five by IVF) and matched parental peripheral blood samples (*n* = 20). The average sequencing yield was 21.01 Gb for neonatal UCB, 20.66 Gb for MPB, and 17.61 Gb for PPB, with all samples covering more than 1 million CpG sites. Clinical characteristics were comparable between the two groups (Tables 1, 2 and 3).

**Table 1:** Baseline characteristics of maternal and newborn variables in IVM and conventional IVF groups

Characteristics	IVM	Conventional IVF	Mean Differences (95% CI)/ Risk Difference (95% CI)	t	*P* value
Paternal age, y	31.00 ± 3.53	30.20 ± 3.63	0.80(-4.42 to 6.02)	0.653	0.733
Maternal age, y	29.60 ± 2.30	29.00 ± 4.18	0.60(-4.32 to 5.52)	0.281	0.786
Maternal BMI, kg/m2	23.48 ± 1.53	22.88 ± 4.18	0.60(-3.99 to 5.19)	0.302	0.771
Basal follicle-stimulating hormone, mIU/ml	5.31 ± 1.52	5.99 ± 1.62	-0.68(-2.97 to 1.61)	-0.686	0.512
Basal luteinizing hormone, mIU/ml	5.02 ± 2.70	8.18 ± 4.63	-3.15(-8.68 to 2.38)	-1.310	0.225
Basal progesterone, nmol/L	1.00 ± 0.33	1.88 ± 1.46	-0.88(-2.42 to 0.67)	-1.310	0.225
Basal estradiol, pmol/L	143.88 ± 52.37	217.2 ± 46.51	-73.32(-145.56 to -1.08)	-2.340	0.047
Testosterone levels, nmol/L	0.86 ± 0.24	1.41 ± 0.74	-0.55(-1.36 to 0.25)	-1.582	0.152
PRL levels, ng/ml	12.80 ± 7.54	11.89 ± 2.52	0.91 (-7.30 to 9.11)	0.255	0.805
A levels, nmol/L	8.15 ± 3.78	10.87 ± 4.35	-2.73 (-8.67 to 3.22)	-1.058	0.321
AMH levels, ng/ml	7.32 ± 1.56	8.03 ± 4.11	-0.71 (-5.25 to 3.83)	-0.361	0.728
Duration of infertility	3.4 ± 2.19	2.4 ± 1.14	1.00 (-1.55 to 3.55)	0.905	0.392
Diabetes during pregnancy∗					
Yes	2	2	0.0% (-51.8% to 51.8%)		1.00
No	3	3			
Hypertension during pregnancy∗					
Yes	2	2	0.0% (-51.8% to 51.8%)		1.00
No	3	3			
Preterm birth (< 37 wks’ gestation)					
Yes	1	0	20.0% (-28.0% to 56.6%)		1.00
No	4	5			
Gestational age at birth, days	269.4 ± 11.7	277.4 ± 5.2	-8.0 (-22.3 to 6.3)	-1.399	0.216
Newborn birth weight, g	3640 ± 889.80	3480 ± 356.37	160.00 (-828.50 to 1148.50)	0.373	0.719
Newborn birth length, cm	51.00 ± 2.50	50.80 ± 2.00	0.20(-2.57 to 2.97)	0.167	0.872

**Table 2:** Ovarian stimulation and embryo development characteristics in IVM and conventional IVF groups

Characteristics	IVM	Conventional IVF	mean differences (95% CI)	t	*P* value
Total gonadotropin doses (IU)	0.00 ± 0.00	1447.50 ± 294.80	-1447.5 (-1813.54 to -1081.46)	-10.979	0.000
Duration of the follicular phase—days	8.60 ± 1.94	11.0 ± 1.00	-2.4 (-4.66 to -0.14)	-2.449	0.040
Duration of stimulation days (d)	0.00 ± 0.00	9.40 ± 1.14	-9.40 (-10.82 to -7.98)	-18.432	0.000
Trigger day E2 value (pg/ml)	184.67 ± 8.50	12988.80 ± 8194.36	-12804.133 (-24760.16 to -848.10)	-2.620	0.040
Trigger day P value (ng/ml)	1.03 ± 0.26	1.95 ± 0.87	-0.918 (-1.99 to 0.15)	-1.733	0.134
Trigger day LH value (IU/L)	6.13 ± 0.92	1.85 ± 0.66	4.28 (2.93 to 5.64)	7.756	0.000
Sperm concentration (10^6^ per mL)	61.07 ± 25.74	37.48 ± 42.01	23.59 (-27.22 to 74.40)	1.070	0.316
Sperm viability (%)	31.35 ± 19.14	29.83 ± 26.50	1.52 (-32.19 to 35.23)	0.104	0.920
No. of retrieved oocytes	19.80 ± 11.65	14.40 ± 6.84	5.4 (-8.53 to 19.33)	0.894	0.398
No. of mature (MII) oocytes	9.60 ± 6.31	11.40 ± 4.04	-1.8 (-9.52 to 5.92)	-0.537	0.606
No. of 2PN embryos	5.40 ± 2.07	7.40 ± 4.16	-2.0 (-6.79 to 2.79)	-0.962	0.364
No. of D3 top-quality cleavage-stage embryos	4.60 ± 2.30	5.60 ± 2.50	-1.0 (-4.51 to 2.51)	-0.657	0.530
Number of blastocysts	2.20 ± 0.84	3.80 ± 2.17	-1.60 (-4.00 to 0.80)	-1.540	0.182

**Table 3:** Metabolic and Hormonal Profiles in IVM and Conventional IVF Groups

Characteristics	IVM	Conventional IVF	mean differences (95% CI)	t	*P* value
Cholesterol ( mmol/L)	4.51 ± 1.42	5.11 ± 1.03	-0.59(-2.40 to 1.21)	-0.758	0.470
TSH Level (mIU/L)	1.69 ± 0.58	2.13 ± 0.43	-0.448(-1.19 to 0.29)	-1.391	0.202
Triglycerides ( mmol/L)	5.06 ± 5.65	1.36 ± 0.60	3.69 (-2.16 to 9.55)	1.453	0.184
HDL-Cholesterol ( mmol/L)	1.16 ± 0.31	1.39 ± 0.27	-0.24(-0.67 to 0.19)	-1.278	0.237
LDL-Cholesterol ( mmol/L)	2.04 ± 0.34	2.82 ± 0.77	-0.78(-1.71 to 0.16)	-2.069	0.088
Glucose metabolism (OGTT)					
Fasting Glucose, mmol/L	6.10 ± 2.46	4.56 ± 0.48	1.54 (-1.05 to 4.13)	1.372	0.207
Glucose (30 min), mmol/L	11.04 ± 4.40	8.32 ± 1.11	2.72 (-1.96 to 7.40)	1.339	0.217
Glucose (60 min), mmol/L	12.08 ± 7.30	6.84 ± 3.17	5.24 (-2.97 to 13.45)	1.472	0.196
Glucose (120 min), mmol/L	11.12 ± 8.85	6.94 ± 1.26	4.18 (-5.03 to 13.39)	1.046	0.326
Insulin sensitivity					
Fasting insulin, mIU/L	11.33 ± 7.55	12.39 ± 11.11	-1.06(-14.92 to 12.79)	-0.177	0.864
Serum Insulin (30 min), mIU/L	72.23 ± 56.56	159.13 ± 105.33	-86.90(-210.19 to 36.40)	-1.625	0.143
Serum Insulin (60 min), mIU/L	102. 25 ± 97.06	98.10 ± 89.49	4.15 (-132.00 to 140.30)	0.070	0.946
Serum Insulin (120 min), mIU/L	63.07 ± 26.47	131.01 ± 88.70	-67.94(-163.40 to 27.52)	-1.641	0.139

**Fig. 1:**
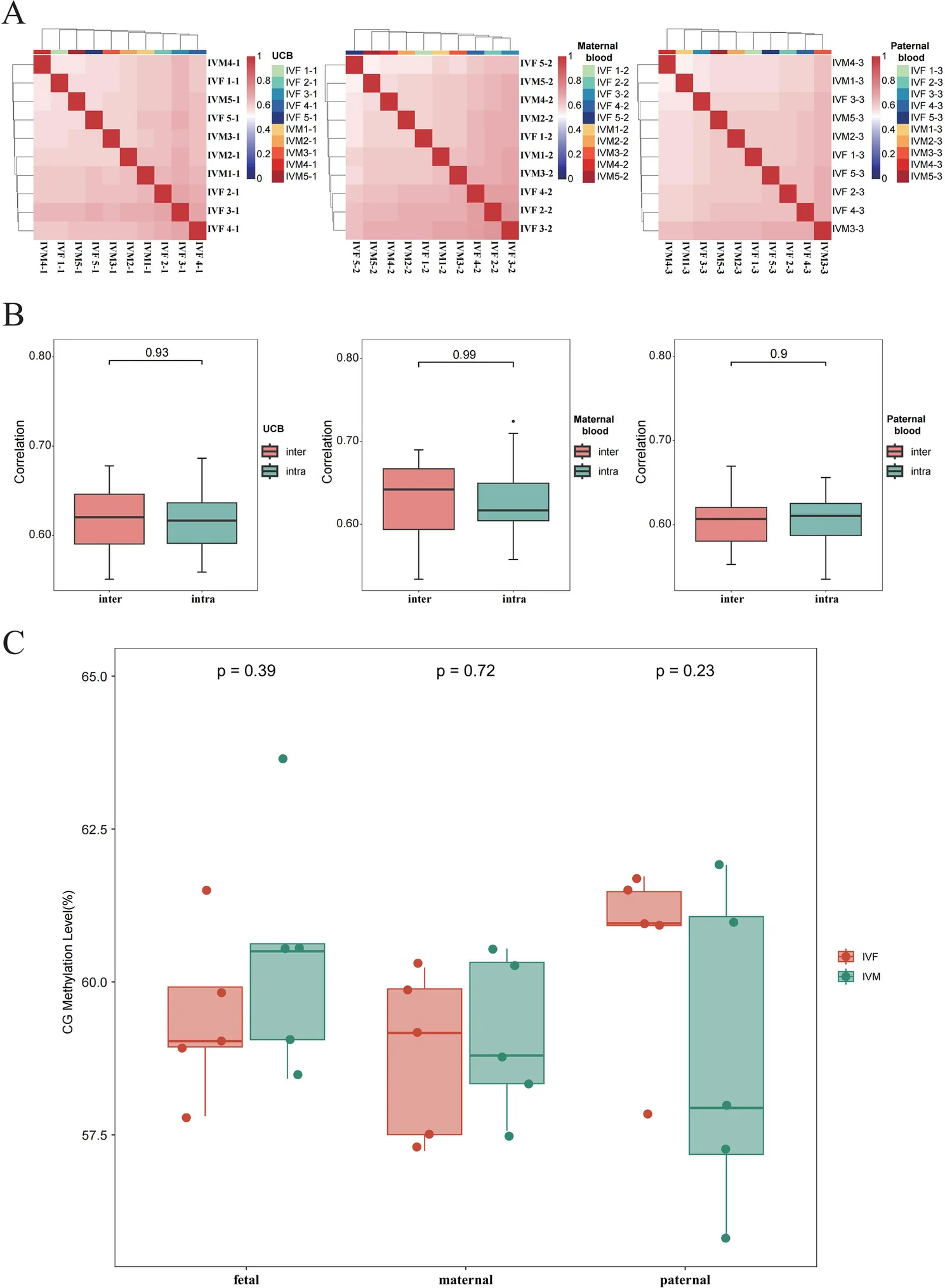
Whole-genome DNA methylation profiling in offspring and parents after IVM versus IVF. **A **Pearson correlation heatmaps of CpG methylation profiles for umbilical cord blood (UCB) from offspring, maternal peripheral blood (MPB), and paternal peripheral blood (PPB). Samples are labelled by family and group (IVF vs IVM). Note the lack of distinct clustering by conception mode (IVM vs IVF) in the UCB samples, indicating that IVM does not induce a systematic, technique-specific alteration in the global methylome. **B **Boxplots of pairwise Pearson correlation coefficients within each tissue type (UCB, MPB, PPB), comparing intra-group correlations (within IVF or within IVM) versus inter-group correlations (between IVF and IVM). **C **Boxplots of genome-wide average CpG methylation level (%) for UCB, MPB, and PPB in the IVF and IVM groups; each dot represents one individual sample (n = 5 per group). Two-sided P values (ANOVA) are shown. The comparable average methylation levels highlight the maintenance of global epigenetic stability in IVM offspring

Heatmap analysis of methylation patterns revealed no significant differences in genome-wide CpG methylation levels between UCB samples from the IVM and IVF groups (Fig. 1A). Similarly, high concordance were observed in paired MPB and PPB samples. Correlation analysis showed moderate Pearson correlation coefficients (*r* = 0.40–0.69) between the groups, suggesting strong consistency in methylation profiles. Further statistical analysis, including boxplot analysis of Pearson correlation coefficients for CpG methylation levels, confirmed no significant differences in either within- or between-group comparisons (Fig. 1B and C). These findings indicate that global CpG methylation patterns were highly correlated and that no extensive epigenetic changes were detected.

Specifically, genome-wide mean CpG methylation levels in IVM-derived UCB samples were not significantly different from those in IVF-derived UCB (*p* > 0.05, ANOVA). Although the IVM group exhibited slightly higher average methylation levels, this difference did not reach statistical significance, likely due to the small sample size. Additionally, methylation variability was slightly greater in the IVM group, but this variation was not statistically significant.

### Methylation analysis across genomic features

**Fig. 2:**
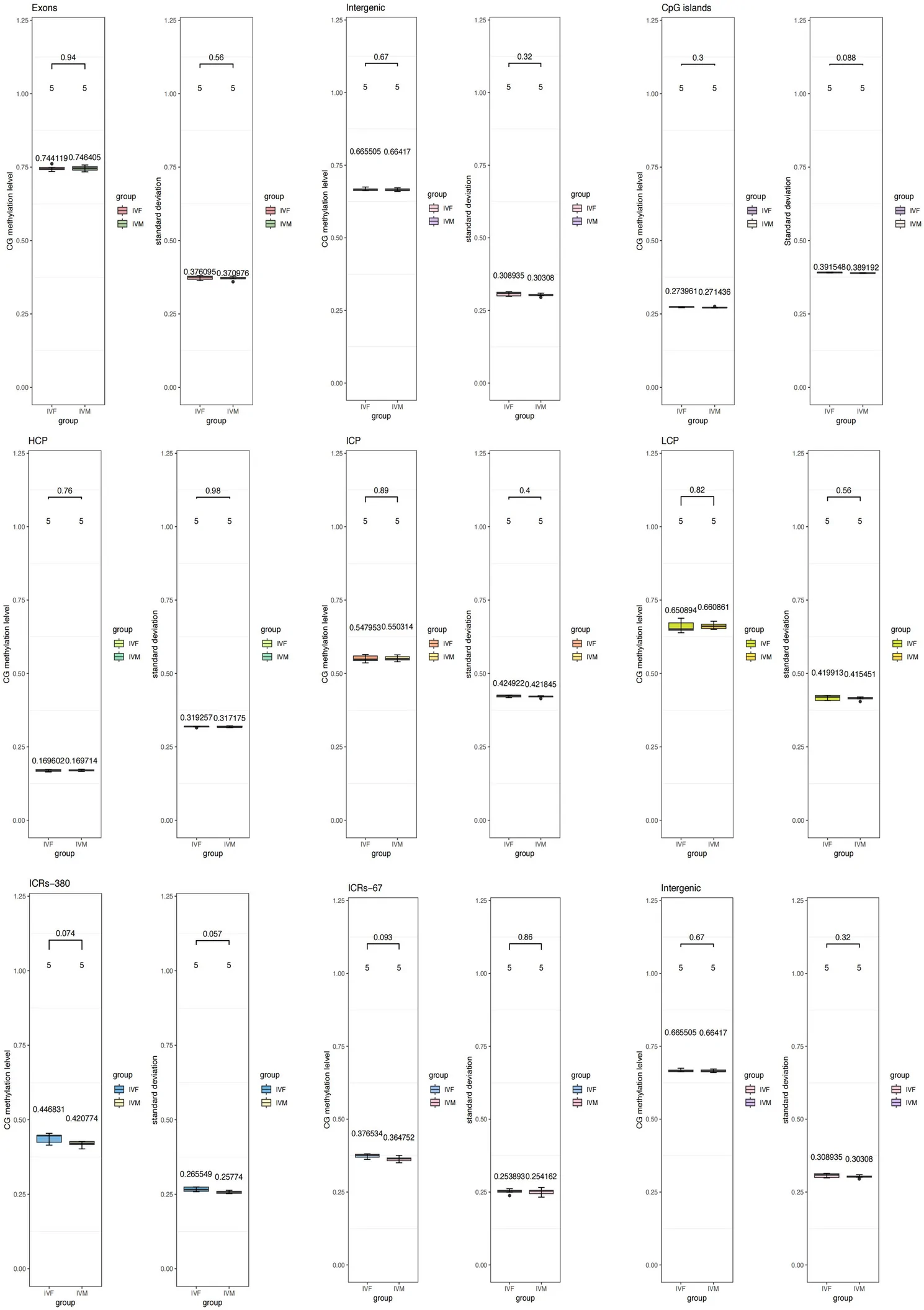
CpG methylation level and variability across functional genomic regions and imprinting-related elements in UCB. Boxplots compare mean CpG methylation level (left panels) and methylation variability (standard deviation; right panels) between IVF and IVM offspring across key genomic annotations, including exons, intergenic regions, CpG islands, promoter CpG-density classes (HCP/ICP/LCP), and imprinting control region sets (ICRs-380 and ICRs-67) (definitions as in Methods). Each dot denotes one UCB sample (n = 5 per group). Two-sided P values for group comparisons are shown

**Fig. 3:**
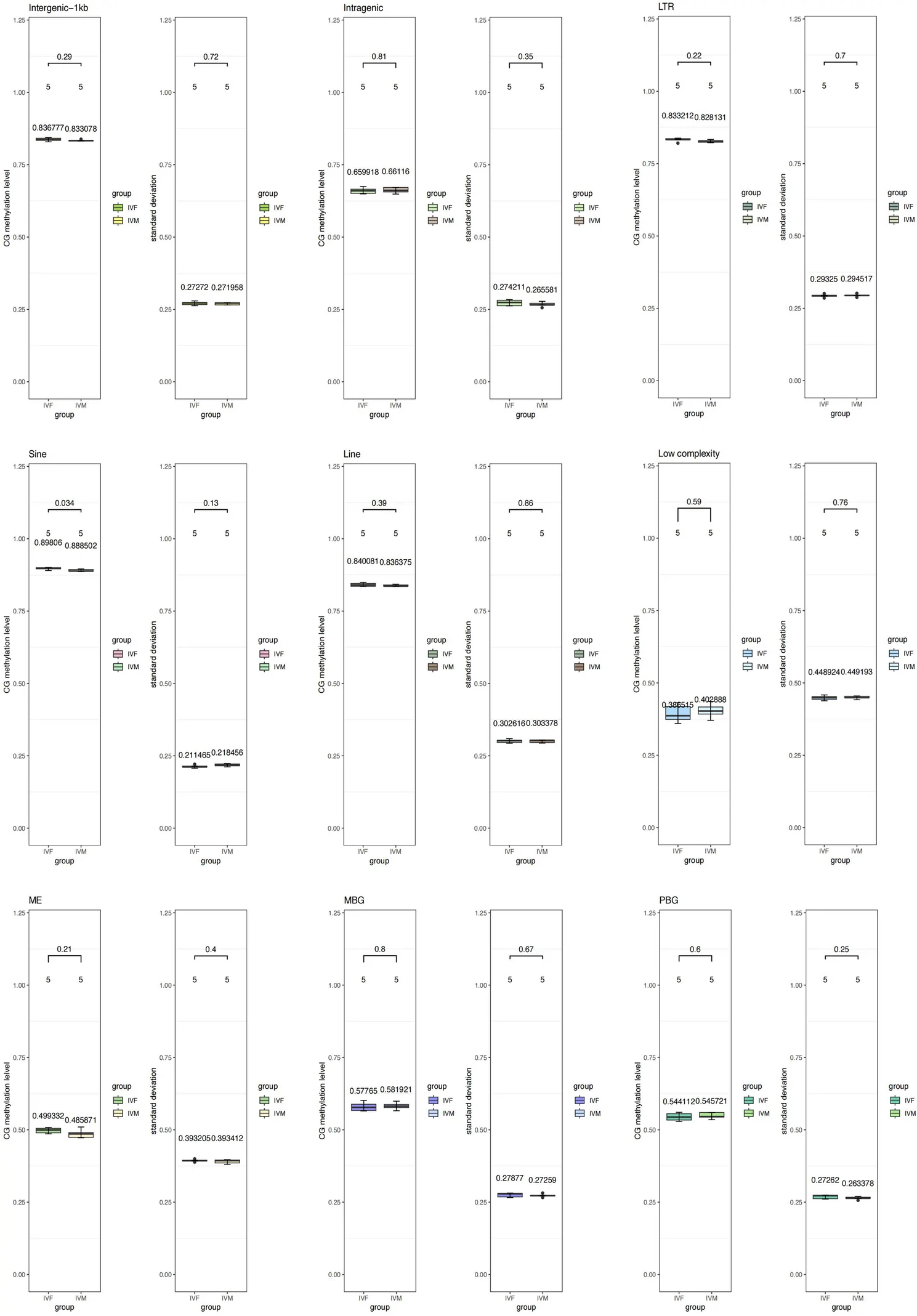
CpG methylation level and variability across additional genomic features and repeat-associated annotations in UCB. Boxplots compare mean CpG methylation level (left panels) and methylation variability (standard deviation; right panels) between IVF and IVM offspring across intergenic flanking regions (Intergenic-1 kb), intragenic regions, major repeat classes (LTR, SINE, LINE, low complexity), and additional annotation tracks (ME, MBG, PBG; definitions as in Methods)

**Fig. 4:**
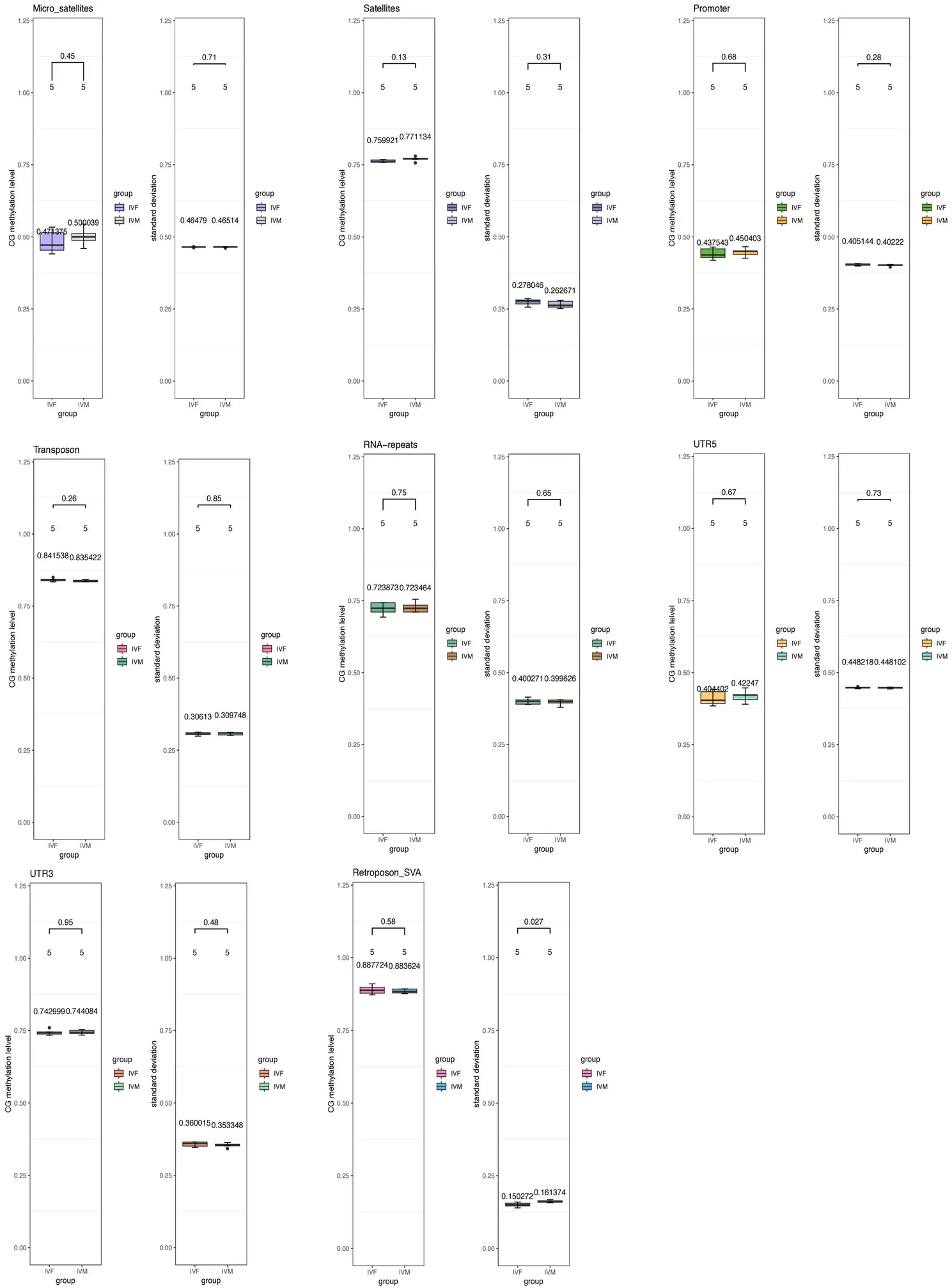
CpG methylation level and variability in repeat subclasses and untranslated regions in UCB. Boxplots compare mean CpG methylation level (left panels) and methylation variability (standard deviation; right panels) between IVF and IVM offspring across repeat subclasses and genic elements, including micro-satellites, satellites, promoters, transposons, RNA repeats, 5′ untranslated regions (UTR5), 3′ untranslated regions (UTR3), and retroposon SVA (Retroposon_SVA)

We further analyzed methylation in annotated genomic regions of neonatal UCB samples, focusing on promoters, exons, introns, intergenic regions, and repetitive elements (such as LINEs, SINEs, and LTRs). The analysis included CpG islands, high- (HCP), intermediate- (ICP), and low-density promoters (LCP), transposon elements, RNA repeats, and imprinting control regions (ICRs). Comparative analysis showed no significant differences in methylation between the IVM and IVF groups across most genomic features (*p* > 0.05, ANOVA) (Figs. 2, 3 and 4).

Furthermore, detailed per-sample distributions across these diverse functional and structural annotations corroborated this high degree of concordance across all individual samples (Figure S1, Figure S2, Figure S3, Figure S4).

Regarding repetitive elements, a marginal statistical reduction in methylation was observed in SINE elements in the IVM group compared to IVF (*p* < 0.05) (Fig. 3).

However, this difference was isolated and small in magnitude. Crucially, it was not accompanied by broad hypomethylation in other major retrotransposons (such as LINEs or LTRs) or increased methylation variability. Given that SINEs are ubiquitous and often serve as surrogates for global methylation maintenance, this isolated marginal fluctuation—without concurrent deregulation of promoters or imprinted loci—is unlikely to represent a functional defect in genomic stability or have clinical consequences.

### Differentially Methylated Regions (DMRs)

**Fig. 5:**
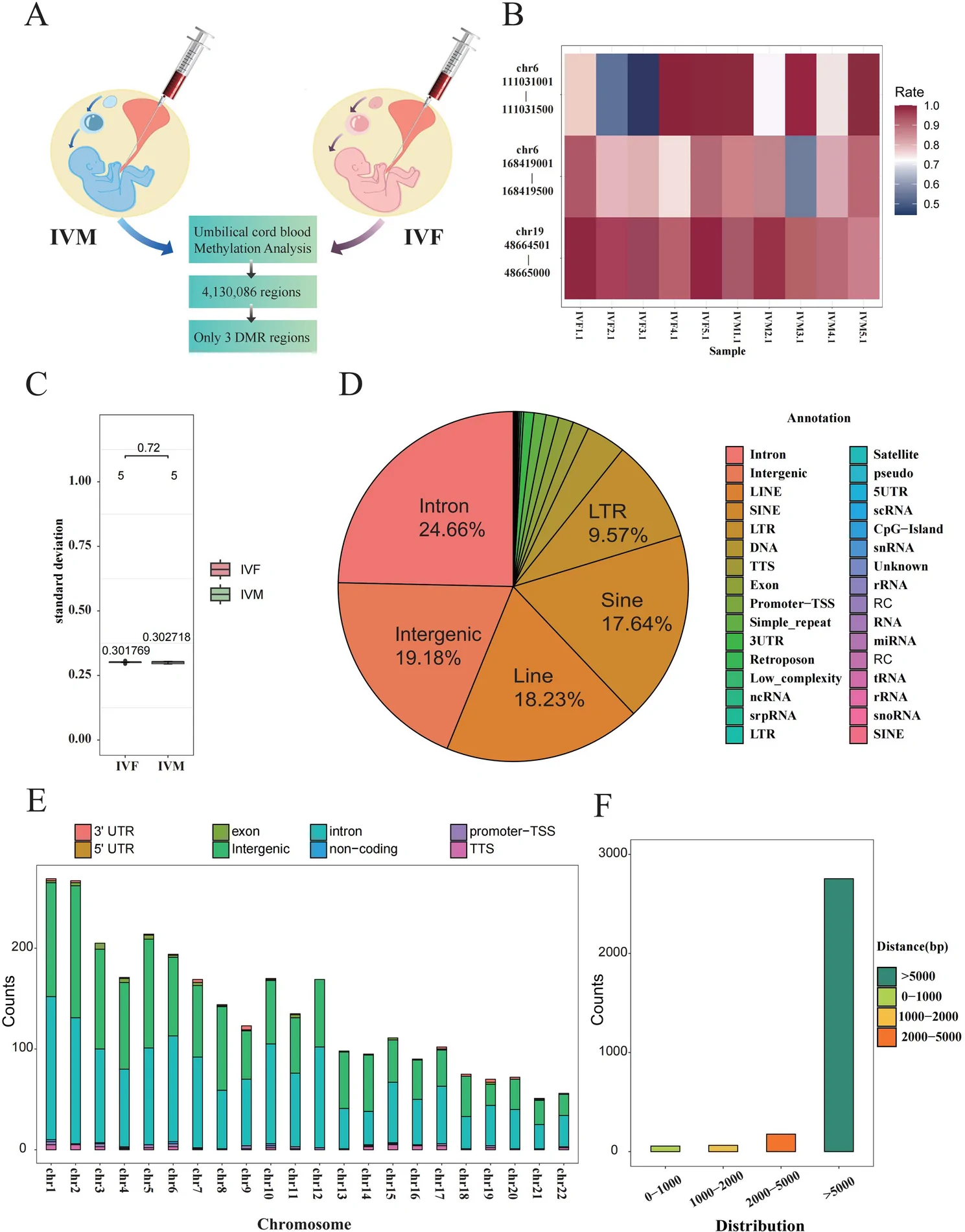
Genome-wide comparison of differential methylation and methylation variability between IVM and IVF offspring. **A **Schematic overview of the region-based workflow, indicating the total number of non-overlapping 500-bp tiles interrogated and the number of differentially methylated regions (DMRs) detected between IVF and IVM groups in UCB. The detection of only 3 DMRs out of >4 million tiles (0.0001%) suggests that the *in vitro* maturation environment has a negligible impact on site-specific methylation fidelity. **B** Heatmap of CpG methylation levels across the identified DMRs, with samples grouped (IVF vs IVM). Colour scale indicates methylation proportion. **C** Boxplot comparing methylation variability (standard deviation, SD) of CpG methylation levels across 500-bp tiles between IVF and IVM offspring in UCB. Mean SD values and the two-sided P value for group comparison are shown. **D**-**F** Characterisation of regions exhibiting discordant variability(defined as high-variability in IVM and low-variability in IVF). **D** Pie chart showing the functional annotation of these overlapping regions; the majority are located in non-coding areas such as introns (24.66%), intergenic regions (19.18%), and LINE elements. **E **Chromosomal distribution of these variable regions. **F** Bar plot illustrating the distribution of these regions relative to the distance from transcription start sites (TSS), showing a predominance in distal regions (>5 kb)

We conducted pairwise comparisons of IVM and IVF samples using 500-bp bins to identify DMRs. To ensure robust identification of biologically relevant differences, we applied stringent statistical criteria (absolute methylation difference ≥ 20% and q-value ≤ 0.05). Among 4,130,086 genomic tiles interrogated, only three DMRs were identified in UCB samples from the IVM group compared to IVF (0.0001% of the total genome) (Fig. 5A and B). These included one hypermethylated region (chr6:111031001–111031500) and two hypomethylated regions (chr6:168419001–168419500 and chr19:48664501–48665000).

This extreme scarcity of DMRs provides strong evidence that the IVM procedure does not induce widespread epigenetic reprogramming errors. Notably, the hypomethylated region on chromosome 19 was located in a gene body associated with NTN5, but no DMRs mapped to known imprinted genes or critical developmental regulators. The absence of perturbations in these clinically sensitive loci suggests that the long-term risk of imprinting disorders or developmental anomalies linked to DNA methylation is likely minimal in IVM offspring.

### Methylation variability analysis

To explore the stability of DNA methylation in IVM versus IVF offspring, we assessed methylation variability by calculating the standard deviation (SD) of methylation levels within 500-bp bins across the genome. A total of 739,154 bins with complete methylation data across all 10 UCB samples were included in the analysis.

High-variability regions were defined as the top 10% of bins in the IVF group and the bottom 10% in the IVM group, resulting in 6,239 overlapping bins. Statistical analysis revealed no significant difference in mean methylation levels between the groups in these high-variability regions (*p* > 0.05, ANOVA) (Fig. 5C).

Functional annotation of the 6,239 high-variability bins showed that 24.66% were located in introns, 19.18% in intergenic regions, and 18.23% in LINE elements. 

Mapping these regions across chromosomes indicated a largely random distribution, predominantly in non-coding regions such as introns and intergenic areas. Notably, 89.66% of these bins were located more than 5 kb away from transcription termination sites, indicating that methylation variability is more prevalent in regulatory regions rather than coding sequences (Fig. 5D, E and F).

Table 1 presents the baseline characteristics of the maternal and paternal populations. No significant differences were found in maternal age, paternal age, BMI, or other baseline characteristics between the two groups. However, basal estradiol levels were significantly lower in the IVM group compared to the conventional IVF group (*P* = 0.047).

Table 2 shows the ovarian stimulation and embryo development parameters. The IVM group required significantly lower total gonadotropin doses and fewer days of stimulation compared to the conventional IVF group (*P* < 0.001). Moreover, trigger day LH levels were significantly higher in the IVM group, while estradiol levels on the trigger day were significantly lower (*P* = 0.040).

Table 3, which includes metabolic and hormonal data, no significant differences were found.

## Discussion

In this trio-based epigenetic sub-study nested within our previously reported RCT of women with PCOS, we found no evidence that IVM is associated with large-scale alterations in offspring cord-blood DNA methylation compared with conventional IVF.

Global CpG methylation levels, methylation patterns across major genomic annotations, and overall methylation variability were comparable between groups. Only three DMRs were identified using stringent thresholds, none of which mapped to established imprinting control regions or clearly developmentally critical loci. 

Although a marginal difference in average methylation across SINE elements was observed, this signal was isolated and not accompanied by broader repeat-element or promoter-associated changes. Collectively, these findings provide the mechanistic safety evidence that complements our 2022 clinical report, confirming that changing the oocyte maturation strategy—from ex vivo maturation of oocytes retrieved from small follicles to in vivo gonadotropin-stimulated maturation—does not introduce detectable systemic methylation perturbations in neonatal cord blood in this PCOS cohort.

Our findings resonate with the emerging consensus in the broader ART epigenetic landscape [11]. While early studies raised concerns about widespread imprinting disruptions, recent large-scale epigenome-wide association studies (EWAS) in IVF cohorts suggest that methylation variations, when present, are typically modest in magnitude, locus-specific, and often resolve over time [14, 19, 20]. Our study extends this reassuring narrative specifically to IVM. Unlike previous candidate-gene studies that focused on isolated imprinted loci, our genome-wide approach confirms that the IVM procedure does not induce “hidden” widespread methylation noise that might have been missed by lower-resolution techniques. Furthermore, our results align with recent EPIC array data from CAPA-IVM cohorts [26], collectively challenging the historical theoretical concern that the prolonged *in vitro* culture duration or the specific *in vitro* maturation medium would inevitably compromise the epigenetic fidelity of the oocyte.

Human epigenetic data directly evaluating IVM remain comparatively sparse but are generally reassuring. Candidate-gene and imprinting-focused analyses in placental tissue, cord blood or children conceived after IVM have not shown increased imprinting error rates at key loci compared with conventional IVF/ICSI [27]. 

Experimental and embryo-stage transcriptomic studies indicate that *in vitro* maturation and related culture conditions can influence pathways linked to oxidative stress, amino acid and one-carbon metabolism and epigenetic enzyme regulation [11, 16, 17]. Together with methylome profiling of human *in vitro* matured oocytes [18], these data underscore the sensitivity of early developmental programming to the *in vitro* environment. However, our findings suggest that such perturbations do not commonly translate into stable, genome-wide DNA methylation changes detectable in neonatal cord blood.

From a clinical management perspective, these findings address a critical bottleneck in the adoption of IVM. For clinicians managing women with PCOS, the decision to choose IVM has historically involved a trade-off: eliminating the immediate physical risk of OHSS at the cost of uncertainty regarding long-term offspring safety. While recent clinical follow-ups have reported reassuring obstetric and long-term developmental outcomes for children conceived via IVM [28], high-resolution molecular safety evidence has been lacking. Our data substantially mitigates this uncertainty. By demonstrating that IVM offspring possess a neonatal methylome highly concordant with that of conventional IVF offspring, this study suggests that the “epigenetic cost” of avoiding OHSS is negligible. Consequently, for high-responder PCOS patients, epigenetic safety concerns should no longer be a primary barrier to recommending IVM, allowing clinicians to prioritize maternal safety and patient comfort without fear of compromising the offspring’s early developmental programming.

This study has several strengths relevant to causal inference within ART. By embedding methylation profiling within a randomised trial restricted to women with PCOS, we reduced confounding by indication and controlled for a PCOS-related endocrine–metabolic background that may influence offspring methylation profiles [22]. Both trial arms used extended culture to the blastocyst stage and a freeze-only single-blastocyst transfer policy, minimising confounding by fresh transfer, multiple gestation or differences in endometrial preparation. The trio-based sampling framework is a critical methodological asset. Although the sample size is limited, this trio-based design significantly enhances our statistical power to detect de novo methylation errors by strictly controlling for parental genetic background, which is often the largest confounder in epigenetic studies. Finally, RRBS provides quantitative methylation estimates across CpG-rich regions and, together with conservative filtering and DMR thresholds, offers robust sensitivity to large-effect changes in promoters and other regulatory features.

Several limitations should be acknowledged. First, the small number of nuclear families limits statistical power to detect subtle effect sizes and reduces the ability to identify rare locus-specific events; therefore, our findings primarily argue against large-scale methylome perturbations rather than establishing equivalence. Second, this epigenetic sub-study relied on the availability of complete parent–offspring trios with biospecimens of sufficient quantity and quality for high-depth RRBS, which resulted in an availability-driven subset of the parent trial cohort. Although selection was based on biospecimen completeness and technical quality rather than clinical outcomes, selection bias cannot be excluded and generalisability may be limited.

Third, umbilical cord blood is a heterogeneous tissue and bulk profiling may obscure cell-type-specific differences. Fourth, we acknowledge the absence of transcriptomic data. However, given the remarkable stability of the DNA methylome observed in our study (with negligible DMRs), the biological impetus to screen for methylation-driven gene expression changes is low. Furthermore, DNA methylation represents a stable “archive” of early developmental impacts, whereas gene expression is transient and highly susceptible to immediate stress during delivery, making methylation a more robust marker for this safety evaluation. Finally, methylation was assessed only at birth, and longer-term follow-up with larger cohorts and additional tissues will be required to determine whether any subtle differences emerge or persist over time.

In conclusion, our trio-based genome-wide analysis provides robust mechanistic evidence supporting the epigenetic safety of IVM. We demonstrate that the distinct oocyte maturation environment of IVM does not lead to systematic or widespread DNA methylation perturbations in PCOS offspring compared to conventional IVF. 

These findings support the clinical utility of IVM as a safe, viable alternative for women with PCOS, validating a treatment strategy that effectively eliminates OHSS while maintaining epigenetic fidelity. While larger longitudinal cohorts are warranted to monitor long-term health, our data offer immediate and robust evidence to guide clinical counseling and support the broader implementation of IVM in reproductive medicine. 

## Supplementary Information

Supplementary Material 1: Figure S1. Per-sample distributions of CpG methylation levels across core functional regions and imprinting-related elements in UCB. For each UCB sample, boxplots show the distribution of CpG methylation levels across 500-bp tiles overlapping exons, introns, CpG islands, promoter CpG-density classes (HCP/ICP/LCP), and imprinting control region sets (ICRs-380 and ICRs-67) (definitions as in Methods). Samples are ordered by group (IVF vs IVM) and family. Numbers above boxplots indicate the number of tiles contributing to each feature for that sample.

Supplementary Material 2: Figure S2. Per-sample distributions of CpG methylation levels across genic context and major repeat classes in UCB.For each UCB sample, boxplots show the distribution of CpG methylation levels across tiles annotated as intergenic, intergenic–1 kb, intragenic, and major repeat classes (LTR, SINE, LINE, low complexity) and ME (definitions as in Methods).

Supplementary Material 3: Figure S3. Per-sample distributions of CpG methylation levels across repeat subclasses and additional annotation tracks in UCB. For each UCB sample, boxplots show the distribution of CpG methylation levels across tiles overlapping MBG, PBG, RNA repeats, micro-satellites, satellites, promoters, transposons, and Retroposon_SVA (definitions as in Methods)

Supplementary Material 4: Figure S4. Per-sample distributions of CpG methylation levels in untranslated regions in UCB. For each UCB sample, boxplots show the distribution of CpG methylation levels across tiles annotated as UTR5 and UTR3 (definitions as in Methods).

Supplementary Material 5.

## Data Availability

The datasets used in the current study are available from the corresponding author upon reasonable request.

## Abbreviations

ART: Assisted reproductive technology
COCs: Cumulus–oocyte complexes
COH: Controlled ovarian hyperstimulation
DMRs: Differentially methylated regions
GnRH: Gonadotrophin-releasing hormone
hCG: Human chorionic gonadotrophin
ICSI: Intracytoplasmic sperm injection
IVF: In vitro fertilization
IVM: In vitro maturation
MII: Metaphase II
MPB: Maternal peripheral blood
OHSS: Ovarian hyperstimulation syndrome
PCOS: Polycystic ovary syndrome
PPB: Paternal peripheral blood
RCT: Randomized controlled trial
RRBS: Reduced representation bisulfite sequencing
UCB: Umbilical cord blood
